# Genomic Insights into Chromosomal Colistin Resistance and Virulence–Resistance Convergence in MDR/XDR *Klebsiella pneumoniae* from Tertiary Hospitals in Peshawar, Pakistan

**DOI:** 10.3390/pathogens15020218

**Published:** 2026-02-14

**Authors:** Aiman Waheed, Sumera Afzal Khan, Sajjad Ahmad, Jody E. Phelan, Gulab Fatima Rani, Susana Campino, Taj Ali Khan, Taane G. Clark

**Affiliations:** 1 Center of Biotechnology and Microbiology, University of Peshawar, Peshawar 25120, Pakistan; draimanwaheed.ibms@kmu.edu.pk (A.W.); drsumera@uop.edu.pk (S.A.K.); 2 Institute of Pathology and Diagnostic Medicine, IPDM, Khyber Medical University, Peshawar 25100, Pakistan; sajjadahmad.ibms@kmu.edu.pk (S.A.); ranigulabfatima@kmu.edu.pk (G.F.R.); 3 Department of Infection Biology, Faculty of Infectious and Tropical Diseases, London School of Hygiene and Tropical Medicine, London WC1E 7HT, UK; jody.phelan@lshtm.ac.uk (J.E.P.); susana.campino@lshtm.ac.uk (S.C.); 4 Public Health Reference Laboratory, Khyber Medical University, Peshawar 25100, Pakistan; 5 Faculty of Epidemiology and Population Health, London School of Hygiene and Tropical Medicine, London WC1E 7HT, UK

**Keywords:** *Klebsiella pneumoniae*, multidrug resistant, extensively drug resistant, whole genome sequencing, chromosomal resistance, colistin resistance, resistome, virulome, plasmids

## Abstract

**Background**: Klebsiella pneumoniae is a World Health Organization-listed critical priority pathogen and a major cause of healthcare-associated infections, driven by the global emergence of multidrug-resistant (MDR) and extensively drug-resistant (XDR) lineages and their alarming convergence with hypervirulence. **Methods**: In this study, 152 clinical specimens, including urine, blood, pus, wound swabs, and respiratory samples, were collected from tertiary care hospitals in Peshawar, Pakistan. Standard microbiological and biochemical methods identified 55 *K. pneumoniae* isolates. Antimicrobial susceptibility testing (AST) was performed using the Kirby–Bauer disk diffusion and broth microdilution methods, with results interpreted according to Clinical and Laboratory Standards Institute (CLSI) guidelines. MDR and XDR phenotypes were defined based on European Centre for Disease Prevention and Control (ECDC) criteria. Whole-genome sequencing (WGS) was conducted on 16 phenotypically confirmed MDR/XDR isolates, followed by comprehensive bioinformatic analyses to characterize sequence types (STs), acquired antimicrobial resistance genes, resistance-associated chromosomal mutations, virulence determinants, plasmid replicons, and phylogenetic relationships. **Results**: Among 55 confirmed *K. pneumoniae* isolates, 19 (34.5%) were classified as MDR and 10 (18.2%) as XDR. WGS revealed substantial genomic heterogeneity, identifying 11 distinct STs, with ST39 being the most prevalent. Resistance to multiple antibiotic classes was mediated by the combined presence of plasmid-borne carbapenemases and extended-spectrum β-lactamases, alongside chromosomal mutations affecting outer membrane porins (*OmpK35*/*OmpK36*), fluoroquinolone targets (*gyrA*/*parC*), efflux regulation (*ramR*, *marR*), and lipid A modification pathways associated with colistin resistance (*mgrB*, *pmrA*/*pmrB*, *arnC*, *crrB*). IncF-family plasmids predominated and frequently co-occurred with additional resistance-associated replicons. Notably, one isolate exhibited an expanded virulence gene repertoire, including multiple siderophore systems and a complete type II secretion system, consistent with a hypervirulence-associated genomic profile. Phylogenetic analyses demonstrated close relatedness to international lineages from Asia, the Middle East, and Europe, indicating regional and transnational dissemination. **Conclusions**: This study highlights the complex interplay between plasmid-mediated gene acquisition and chromosomal adaptive mutations driving MDR and XDR phenotypes in *K. pneumoniae* circulating in Peshawar, Pakistan. The identification of hypervirulence-associated genetic features within an MDR background underscores the growing threat posed by convergent lineages and emphasizes the need for sustained WGS-based surveillance to inform infection control and antimicrobial stewardship strategies.

## 1. Introduction

*Klebsiella pneumoniae* is a Gram-negative opportunistic pathogen and a leading cause of severe community and hospital-acquired infections, including pneumonia, bloodstream infections, urinary tract infections, wound infections, and intra-abdominal sepsis [1]. Its clinical importance is particularly pronounced in hospitalized, immuno-compromised, and critically ill patients, where infections are associated with prolonged hospitalization and high mortality rates [2]. Over the past two decades, the global burden of *K. pneumoniae* infections has increased substantially, largely driven by the rapid emergence and dissemination of antimicrobial resistance (AMR) [3].

The World Health Organization (WHO) has classified carbapenem-resistant *K. pneumoniae* as a critical priority pathogen, reflecting the severe therapeutic challenges posed by resistance to last-line antibiotics [4,5]. Resistance in *K. pneumoniae* is mediated through a multifaceted array of mechanisms, including the acquisition of plasmid-encoded carbapenemases and extended-spectrum β-lactamases, alterations in outer membrane permeability, overexpression of multidrug efflux systems, and chromosomal mutations in antibiotic targets and regulatory pathways [6,7]. Particularly concerning is resistance to colistin, a last-resort antimicrobial, which is increasingly driven by chromosomal alterations affecting lipid A biosynthesis and two-component regulatory systems rather than transferable *mcr* genes [8,9].

In parallel with escalating resistance, *K. pneumoniae* exhibits a diverse arsenal of virulence factors that enhance colonization, immune evasion, and pathogenicity. These include a polysaccharide capsule, fimbrial adhesins, iron-acquisition systems, and specialized secretion systems [10]. Hypervirulent *K. pneumoniae* lineages, traditionally associated with community-acquired invasive infections, are characterized by expanded siderophore repertoires and enhanced capsule regulation [11]. Alarmingly, recent genomic studies have documented the convergence of MDR and hypervirulence within the same lineages, generating strains with both enhanced pathogenic potential and severely limited treatment options [12].

Whole-genome sequencing (WGS) has emerged as a powerful tool for dissecting the population structure, resistance mechanisms, and virulence profiles of *K. pneumoniae* at high resolution [13]. By enabling simultaneous analysis of STs, resistance genes, chromosomal mutations, plasmid architectures, and phylogenetic relationships, WGS provides critical insights into the evolutionary dynamics and transmission pathways of high-risk clones [14]. Such genomic surveillance is particularly important in low- and middle-income countries, where antibiotic misuse, limited infection control infrastructure, and insufficient molecular surveillance may accelerate the spread of resistant pathogens [15].

Despite increasing reports of MDR *K. pneumoniae* in Pakistan, most existing studies have focused on phenotypic resistance patterns or the detection of selected resistance genes, with limited integration of WGS data. As a result, the genomic architecture underlying antimicrobial resistance in local *K. pneumoniae* populations—particularly the relative contribution of chromosomal adaptive mechanisms to colistin resistance and the extent of virulence–resistance convergence—remains poorly defined, especially in the Peshawar region [12,15,16,17,18,19,20]. This study aimed to evaluate the genomic basis of MDR/XDR *K. pneumoniae*, with particular emphasis on chromosomal colistin resistance and virulence–resistance convergence in Peshawar. To achieve the aim, phenotypic AST was integrated with WGS-based analyses of clonal structure, resistomes, virulomes, plasmid replicon diversity, and phylogenetic relationships, providing comprehensive insight into resistance evolution in an understudied setting.

## 2. Materials and Methods

### 2.1. Study Design, Ethical Approval, and Clinical Sample Collection

This cross-sectional study was conducted using clinical samples (blood, urine, pus, wound swabs, sputum, and other body fluids) collected from patients attending three tertiary care hospitals in Peshawar, Pakistan, namely, Khyber Teaching Hospital (KTH), Lady Reading Hospital (LRH), and Hayatabad Medical Complex (HMC). These hospitals serve as major referral centers for the surrounding districts where appropriate healthcare facilities are not available for microbiological analysis.

The study was approved by the Research Ethics Board of the University of Peshawar (Approval No. REB-10, approved on 12 July 2024). Written informed consent was obtained from all participants or their legal guardians prior to sample collection.

A total of 152 non-duplicate samples were collected between January 2023 and June 2024 from patients with clinical features suggestive of Gram-negative bacterial infection, as assessed by the physicians (e.g., suspected urinary tract infection, bloodstream infection, respiratory infection, wound or soft tissue infection). To minimize confounding by prior antimicrobial exposure, patients who had received systemic antibiotic therapy within 72 h before specimen collection were excluded. All samples were collected aseptically by trained clinical staff, properly labelled, placed in sterile, leak-proof containers and transported to the Microbiology laboratory of the Centre of Biotechnology and Microbiology, University of Peshawar, under standard biosafety conditions within 2–4 h of collection for further processing. When immediate processing was not feasible, specimens were stored at 4 °C and processed within 24 h according to laboratory operating procedures in compliance with WHO laboratory guidelines [21].

### 2.2. Culture-Based Isolation and Identification of K. pneumoniae

Clinical samples were then aseptically inoculated onto MacConkey agar, cystine lactose electrolyte deficient (CLED) agar, and blood agar (Oxoid, UK), followed by incubation at 37 °C for 18–24 h. The *K. pneumoniae* were identified based on their characteristic large, mucoid, lactose-fermenting pink colonies on MacConkey agar, and Gram-negative rod-shaped appearance on Gram staining.

The *K pneumoniae* isolates were characterized using standard biochemical tests, including oxidase, urease, indole production, motility, methyl red, Voges–Proskauer, and citrate utilization assays. The non-motile growth pattern was demonstrated by confinement of bacterial growth to the line of inoculation in Motility indole urea agar medium [22]. Further confirmation was performed using the API 20E system (bioMérieux, Lyon, France) according to the manufacturer’s instructions, with biochemical profiles concordant with the *K. pneumoniae* reference identification code 5214573, as part of routine laboratory screening. Confirmed *K. pneumoniae* isolates were preserved in Luria–Bertani broth supplemented with 40% glycerol and stored at −80 °C until further analysis [23].

Definitive species-level confirmation was subsequently achieved in silico using WGS data. Taxonomic classification of sequenced isolates was performed using Kraken2 (v2.17.1) software, and only isolates confidently assigned to *K. pneumoniae* were included in downstream genomic analyses [24].

### 2.3. Antimicrobial Susceptibility Testing and Phenotypic Classification

AST was performed for all confirmed *K. pneumoniae* isolates using the Kirby Bauer disc diffusion method on Mueller–Hinton agar, following standardized laboratory protocols and broth microdilution methods [25,26]. A panel of 14 clinically relevant antibiotics representing major antimicrobial classes was tested, including β-lactams, aminoglycosides, fluoroquinolones, tetracyclines, glycylcyclines, and carbapenems (Table S1). The diameter of inhibition zones was measured and interpreted according to the Clinical and Laboratory Standards Institute (CLSI) 2022 guidelines [27]. These antimicrobial agents were selected to capture the major classes recommended for treating Enterobacterales infections, thereby ensuring relevance to both international clinical guidelines and local prescribing patterns in tertiary care settings.

Colistin and tigecycline susceptibility were determined using broth microdilution (ComASP™ Colistin, Liofilchem, Italy), with minimum inhibitory concentration (MIC) values interpreted according to CLSI breakpoints [26,28]. The MDR and XDR phenotypes were defined using the European Centre for Disease Prevention and Control (ECDC) criteria based on non-susceptibility across antimicrobial classes rather than the absolute number of agents tested. MDR was defined as non-susceptibility to at least one agent in three or more antimicrobial categories, while XDR was defined as non-susceptibility to all but two or fewer antimicrobial categories tested [29].

### 2.4. Whole-Genome Sequencing

Based on phenotypic resistance profiles and quality of DNA, a subset of 16 isolates exhibiting MDR or XDR phenotypes (9 MDR and 7 XDR) was selected for WGS. This targeted selection was designed to maximize representation of advanced resistance phenotypes while maintaining analytical feasibility.

Genomic DNA was extracted using the GeneJET Genomic DNA Purification Kit (Thermo Fisher Scientific, Waltham, MA, USA) according to the manufacturer’s protocol. DNA quantity and purity were assessed using agarose gel electrophoresis and NanoDrop spectrophotometry (Thermo Fisher Scientific, Wilmington, DE, USA). Paired-end sequencing (2 × 150 bp) was performed on the Illumina MiSeq platform at the Applied Genomics Centre, London School of Hygiene and Tropical Medicine [30].

### 2.5. Bioinformatic Processing and Genome Assembly

Raw sequencing reads were assessed for quality using FastQC (v0.12.1) [31], followed by adapter removal and quality trimming using fastp (v1.1.0) [32]. Quality metrics before and after trimming were summarised using MultiQC (v1.19) [33]. Taxonomic classification was performed using Kraken2 software (v1.3.1), with visualization of outputs using Krona to confirm species identity [24]. High-quality reads were assembled de novo using SPAdes implemented within the Shovill pipeline (v1.4.2). Assembly quality metrics, including genome size, contig number, N50, and L50 values, were evaluated using QUAST. Genome annotation was conducted using both Prokka (v1.14.6) and RAST to identify coding sequences, rRNA, and tRNA genes [34].

### 2.6. Genomic Characterisation of Resistance, Virulence, and Plasmids

Multilocus sequence typing (MLST) was performed using the PubMLST database (v2.23.0) to determine STs and assess clonal diversity [13]. Acquired antimicrobial resistance genes and resistance-associated chromosomal mutations were identified using ResFinder (v4.1), CARD, AMRFinderPlus (v3.12.8), and ABRicate (v1.0.1) [24,35].

Virulence-associated genes were detected using the Virulence Factor Database (VFDB) [35], while plasmid replicon types were identified using PlasmidFinder (v2.1) [34]. All genomic predictions were interpreted conservatively as putative determinants based on sequence homology and curated databases.

### 2.7. Phylogenetic and Comparative Genomic Analyses

Pan-genome analysis was conducted using Prokka-annotated GFF files as input for the Roary pipeline (v3.13.0), defining the core genome at ≥95% protein identity and presence in ≥99% of isolates. Core genome alignments were used to infer maximum-likelihood phylogenies with IQ-TREE (v2.4.0). In parallel, single-nucleotide polymorphism (SNP)-based phylogenetic analysis was performed using the Snippy pipeline (v4.6.0) [34], with alignments inspected using MAFFT and AliView [36]. Phylogenetic trees were visualized and annotated using the Interactive Tree of Life (iTOL) platform (https://itol.embl.de/personal_page.cgi, accessed on 11 February 2026). Comparative phylogenetic analyses included publicly available *K. pneumoniae* genomes to contextualize study isolates within a global framework.

### 2.8. Data Interpretation and Reproducibility

All bioinformatic analyses were conducted using validated pipelines and publicly available databases. Resistance and virulence predictions were interpreted in the context of phenotypic data where applicable. No causal inferences were made without experimental validation, and all genomic associations are reported as putative unless otherwise demonstrated. For antimicrobial susceptibility data, resistance proportions were summarized descriptively, and 95% confidence intervals were calculated using the exact binomial (Clopper–Pearson) method [37].

## 3. Results

### 3.1. Clinical Characteristics of K. pneumoniae

Of the 152 clinical specimens processed, 55 (36.2%) isolates were confirmed as *K. pneumoniae* based on culture characteristics, Gram staining, and API 20E biochemical identification. The median age of infected patients was 33.4 years (range: 21–47 years), with a slightly higher proportion of infections observed in females (54.5%) than males (45.5%).

Urinary tract infections represented the most frequent clinical presentation (23.6%), followed by abscesses (21.8%), skin and soft tissue infections (16.4%), bacteremia (14.5%), respiratory tract infections (12.7%), and wound infections (11.0%). Most patients were admitted to general surgery (27.3%) or internal medicine wards (25.5%), while 14.5% required intensive care unit (ICU) admission, reflecting the clinical severity associated with *K. pneumoniae* infections in this setting (Table S2).

### 3.2. Phenotypic Antimicrobial Susceptibility Profiles

**Figure 1:**
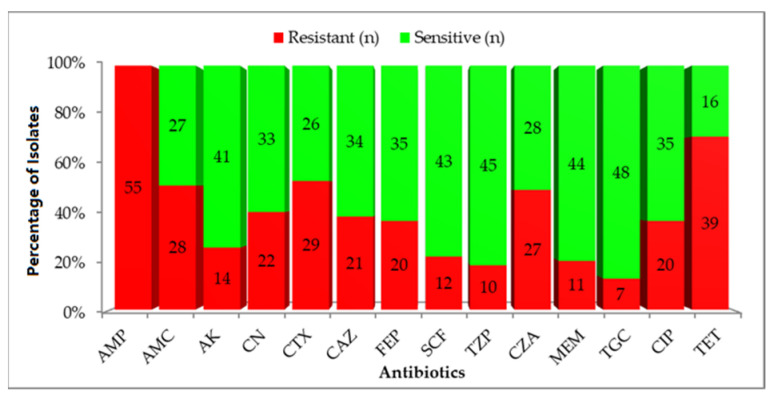
Antibiotics susceptibility patterns of 55 *K. pneumoniae* isolates. AMP, Ampicillin; AMC, Amoxicillin–Clavulanic acid; AK, Amikacin; CN, Gentamicin; CTX, Cefotaxime; CAZ, Ceftazidime; FEP, Cefepime; SCF, Cefoperazone–Sulbactam; TZP, Piperacillin–Tazobactam; CZA, Ceftazidime–Avibactam; MEM, Meropenem; TGC, Tigecycline; CIP, Ciprofloxacin; TET, Tetracycline.

AST demonstrated a high prevalence of resistance across multiple antibiotic classes. All isolates were resistant to ampicillin (100%; 95% CI: 93.5–100), consistent with intrinsic β-lactam resistance. High resistance rates were observed for tetracycline (70.9%; 95% CI: 57.9–81.2), cefotaxime (52.7%; 95% CI: 39.0–66.1), amoxicillin–clavulanate (50.9%; 95% CI: 37.3–64.4), and cefazolin (49.1%; 95% CI: 35.6–62.7), indicating widespread compromise of commonly used antibiotics. Moderate resistance was detected for gentamicin (40.0%; 95% CI: 27.1–54.4), ciprofloxacin (36.4%; 95% CI: 23.8–50.4), ceftazidime (38.2%; 95% CI: 25.4–52.3), and cefepime (36.4%; 95% CI: 23.8–50.4). Resistance to last-line agents remained clinically concerning, with meropenem resistance detected in 20.0% (95% CI: 10.4–33.0) of isolates and colistin resistance identified in 14.5% (95% CI: 6.8–26.6) based on broth microdilution testing. Tigecycline retained the highest activity, with resistance observed in only 12.7% (95% CI: 5.6–24.2) of isolates (Figure 1). Using ECDC definitions, 19 isolates (34.5%; 95% CI: 22.6–48.0) were classified as MDR, and 10 isolates (18.2%; 95% CI: 9.2–30.9) met the criteria for phenotypic XDR, highlighting a substantial burden of advanced resistance in the study population.

### 3.3. Genomic Diversity and Population Structure of MDR/XDR Isolates

WGS was performed on 16 MDR/XDR *K. pneumoniae* isolates selected based on resistance phenotype and DNA quality. Genome assemblies exhibited considerable variability, with genome sizes ranging from 3.99 Mb to 10.24 Mb and predicted coding sequences ranging from 3895 to 10,232, reflecting pronounced genomic plasticity (Table S3).

**Figure 2:**
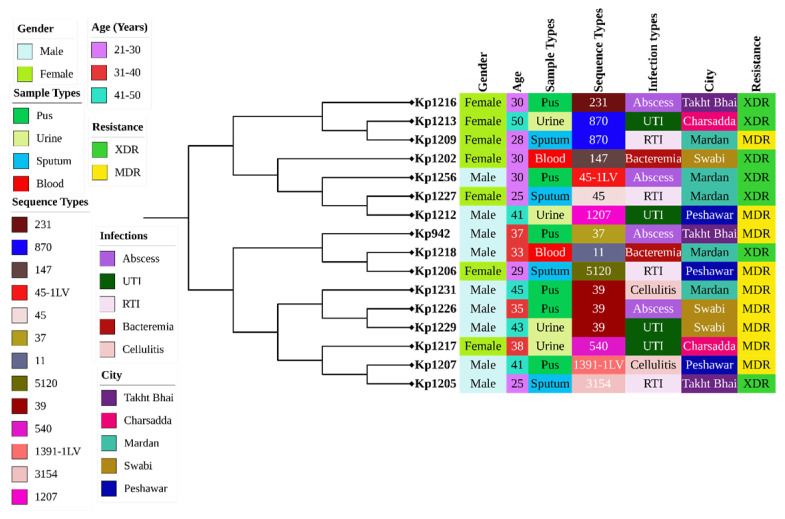
Core-genome phylogenetic tree generated using iTOL, incorporating metadata from 16 *K. pneumoniae* clinical isolates analyzed by WGS.

MLST identified 11 distinct STs, indicating that MDR/XDR phenotypes were distributed across diverse genetic backgrounds. ST39 was the most frequently identified lineage (18.8%), followed by ST870 (12.5%), while the remaining isolates belonged to a range of STs, including internationally recognized high-risk lineages. The identification of single-locus variants in two isolates suggests ongoing microevolution within circulating *K. pneumoniae* populations (Figure 2).

### 3.4. Phylogenomic Relationships and Global Contextualization

**Figure 3:**
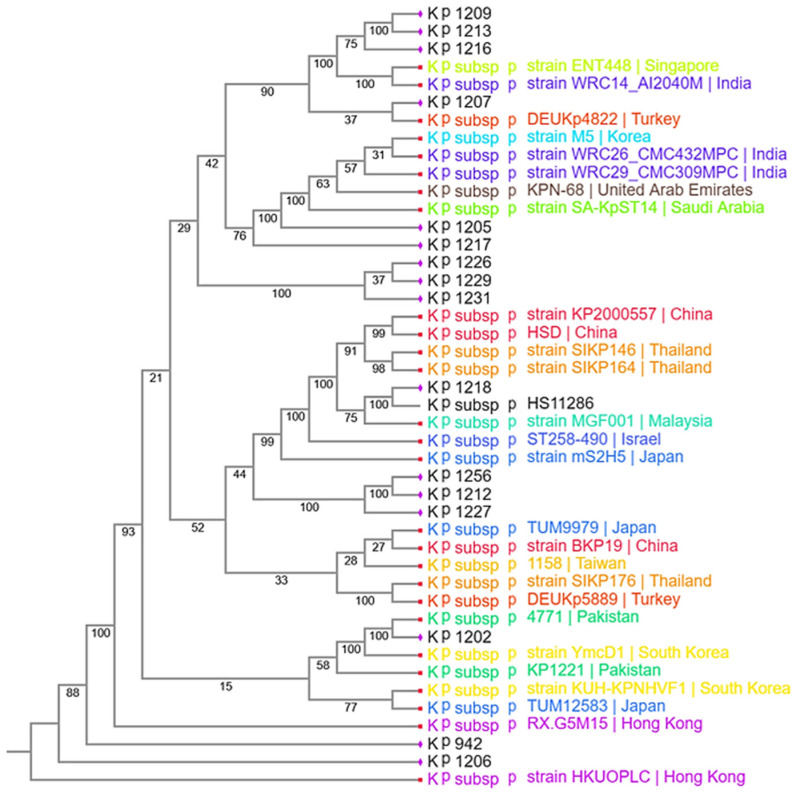
The phylogenetic tree illustrates the evolutionary relationships among 16 *K. pneumoniae* isolates from this study and 27 isolates from various Asian countries. Different colors represent specific countries, while isolates of the present study are labelled in black.

Core genome phylogenetic analysis revealed both clonal clustering within individual STs and broader divergence across lineages. Isolates belonging to ST39 formed a distinct cluster, consistent with clonal expansion accompanied by within-ST diversification. To contextualize the local isolates within a global genomic framework, 27 publicly available *K. pneumoniae* genomes were retrieved from the BV-BRC database. Reference genomes were selected based on their isolation from human clinical infections, availability of high-quality assemblies, and collection between 2010 and 2020, a period corresponding to the global emergence and expansion of MDR/XDR *K. pneumoniae*. This approach was intended to provide temporal and geographic context for phylogenomic comparison rather than to infer a direct epidemiological linkage. Comparative phylogenomic analysis showed that the study isolates clustered with reference genomes originating from Asia, the Middle East, and Europe, indicating that MDR/XDR *K. pneumoniae* circulating in Peshawar, Pakistan, belongs to broader international lineages rather than being restricted to localized evolutionary trajectories (Figure 3).

### 3.5. Integrated Resistome Analysis: Acquired Genes and Chromosomal Adaptation

**Figure 4:**
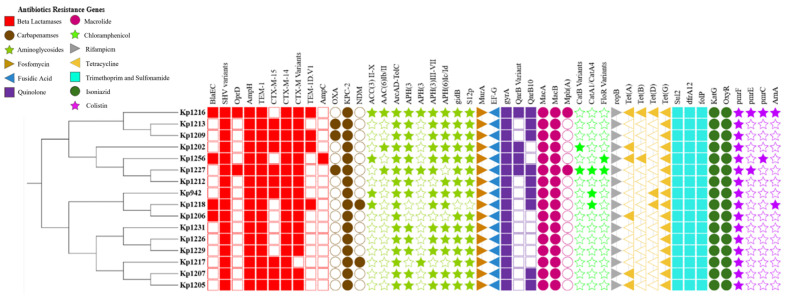
Phylogenetic tree of *K. pneumoniae* core genomes showing the distribution of AMR genes. Filled symbols indicate the presence of drug resistance genes, while empty symbols indicate absence, generated using iTOL.

Comprehensive resistome profiling revealed that MDR was mediated through the combined presence of plasmid-encoded resistance genes and chromosomal adaptive mutations. All sequenced isolates carried the intrinsic *blaSHV* β-lactamase. In addition, a wide array of plasmid-mediated β-lactamases, including extended-spectrum β-lactamases and carbapenemases, was identified, consistent with phenotypic resistance to cephalosporins and carbapenems. Aminoglycoside resistance determinants, plasmid-mediated quinolone resistance genes, and multidrug efflux systems were widely distributed across isolates, supporting resistance to multiple antibiotic classes (Figure 4). Chromosomal mutations affecting outer membrane permeability and regulatory pathways were also prevalent. Alterations in porins *OmpK35* and *OmpK36* were detected in most isolates, frequently involving loop regions implicated in antibiotic influx. Fluoroquinolone resistance was associated with canonical target-site mutations in *gyrA* and *parC*, often accompanied by mutations in global regulators (*ramR* and *marR*), suggesting a combined contribution of target modification and efflux regulation (Table 1). These associations are putative, based on sequence homology and prior literature, and were not functionally validated.

### 3.6. Putative Chromosomal Determinants of Colistin Resistance

Colistin resistance was primarily associated with chromosomal mutations affecting lipid A biosynthesis and regulatory pathways, including *mgrB*, *pmrA*, *pmrB*, *crrB*, *arnC*, *lapB*, and *lpxM*. These genetic changes are consistent with activation of lipid A modification pathways that reduce colistin binding. The mutations in *mgrB* were detected in all isolates, indicating that the presence of such mutations alone is insufficient to infer phenotypic resistance. Consequently, these genetic findings are interpreted as putative resistance-associated features rather than proven causal determinants. Notably, plasmid-mediated *mcr* genes were not detected, indicating that resistance to colistin in this cohort was driven predominantly by chromosomal adaptation rather than horizontal gene transfer (Table 1).

### 3.7. Virulence-Associated Genomic Features and Convergence with MDR

**Figure 5:**
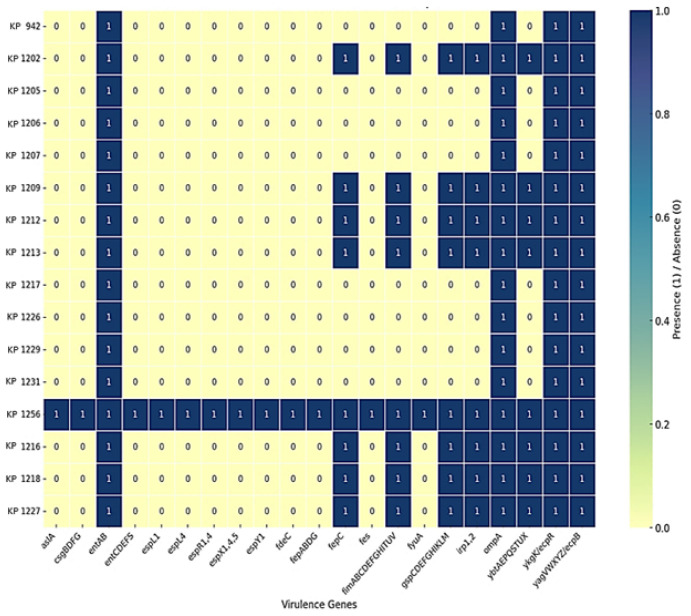
Heatmap showing the virulence genes identified in *K. pneumoniae* using VFDB (absence = yellow, value 0; presence = blue, value 1).

Virulence profiling demonstrated heterogeneity across the sequenced isolates. Core virulence-associated genes, including *ompA* and type 1 fimbrial genes, were conserved across all genomes, supporting baseline adhesive and colonization capacity. A subset of isolates exhibited expanded virulence-associated gene repertoires. One isolate (Kp1256), recovered from a skin abscess sample of a 30-year-old male patient, harbored multiple siderophore systems, immune evasion factors, adhesion determinants, and a complete type II secretion system. Although these genomic features are frequently associated with hypervirulence-linked lineages, phenotypic assays such as the string test, siderophore quantification, or in vivo infection models were not performed; therefore, they are interpreted as genomic indicators of expanded virulence potential rather than confirmed hypervirulence. The coexistence of extensive virulence determinants within an MDR genetic background highlights the potential emergence of convergent high-risk *K. pneumoniae* lineages (Figure 5).

### 3.8. Plasmid Replicon Diversity

**Figure 6:**
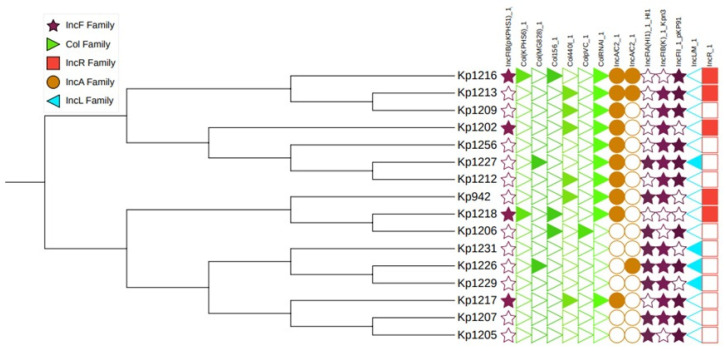
Phylogenetic tree of the current study *K. pneumoniae* isolates (*n* = 16) showing the distribution of plasmid replicon families. Filled symbols indicate the presence and empty symbols indicate absence, generated using iTOL.

Plasmid analysis revealed diverse replicon profiles across MDR/XDR isolates. IncF-family plasmids were ubiquitous and frequently co-occurred with other replicon types, including IncR, IncA/C2, IncL/M, and multiple Col-type plasmids. Several isolates harbored multiple plasmid replicons simultaneously, consistent with the accumulation of resistance determinants on stable plasmid backbones. The predominance of IncF plasmids underscores their central role in the dissemination and maintenance of MDR within clinical *K. pneumoniae* populations (Figure 6).

## 4. Discussion

*K. pneumoniae* represents one of the most critical threats among healthcare-associated bacterial pathogens due to its extraordinary capacity to acquire antimicrobial resistance and its increasing convergence with enhanced virulence [3,38]. In this study, we combined phenotypic antimicrobial susceptibility testing with WGS to provide an integrated characterization of MDR and XDR *K. pneumoniae* isolates circulating in tertiary care hospitals in Peshawar region of Pakistan. Rather than confirming predefined expectations, our study findings reveal substantial genomic heterogeneity, complex multilayered resistance mechanisms, and evidence of virulence-associated genomic features within MDR backgrounds, illustrating the complexity of resistance evolution in this setting.

A key finding of this work is the high prevalence of advanced resistance phenotypes, with more than half of the clinical isolates classified as MDR or XDR. Resistance to first-line and second-line antibiotics was widespread, and clinically concerning resistance to last-line agents, including carbapenems and colistin, was observed. These findings are consistent with, but do not extend beyond, patterns reported in previous phenotypic studies from Pakistan and neighboring regions, which have documented escalating resistance among *K. pneumoniae* isolates [2,39,40,41,42]. However, most existing Pakistani studies rely primarily on phenotypic data or targeted resistance gene detection, limiting direct genomic comparison [43]. Our results therefore complement, rather than supersede, earlier reports by providing genome-resolved insights from an understudied region. Importantly, the persistence of tigecycline activity against most isolates suggests that therapeutic options remain available, although the clinical durability of such last-line agents warrants cautious interpretation [44,45].

WGS revealed pronounced genomic diversity among MDR/XDR isolates, with resistance distributed across multiple STs rather than being driven by a single dominant clone as observed in other studies conducted in India and Africa [46,47]. This observation suggests that resistance in this setting may arise through repeated acquisition of resistance determinants across diverse genetic backgrounds, rather than providing evidence for clonal expansion alone [48]. Phylogenomic analyses further demonstrated that study isolates cluster with international lineages from Asia, the Middle East, and Europe, indicating regional and transnational dissemination of high-risk *K. pneumoniae* lineages. These findings emphasize the interconnected nature of antimicrobial resistance evolution and the need for coordinated surveillance efforts that extend beyond local or national boundaries [5].

At the mechanistic level, our results highlight the combined contribution of plasmid-mediated resistance and chromosomal adaptive mutations to MDR and XDR phenotypes. Plasmid-borne extended-spectrum β-lactamases and carbapenemases were widely distributed, explaining phenotypic resistance to cephalosporins and carbapenems [49]. IncF-family plasmids predominated and frequently co-occurred with additional replicon types, consistent with their established role as stable backbones for the accumulation and dissemination of resistance genes in clinical *K. pneumoniae* populations [50].

In parallel, chromosomal mutations affecting membrane permeability, antibiotic targets, and regulatory pathways were pervasive and likely play a critical role in amplifying resistance phenotypes. Alterations in outer membrane porins *OmpK35* and *OmpK36*, particularly within loop regions associated with antibiotic influx, were detected in the majority of sequenced isolates and are consistent with reduced β-lactam permeability [51,52]. Fluoroquinolone resistance was associated with canonical target-site mutations in *gyrA* and *parC*, often accompanied by mutations in global regulators such as *ramR* and *marR*, suggesting a combined contribution of target modification and efflux regulation [53,54]. These findings underscore the importance of chromosomal adaptation in shaping resistance phenotypes, particularly in highly resistant strains where plasmid-mediated mechanisms alone may be insufficient [49].

Colistin resistance in this cohort was predominantly mediated by chromosomal mutations affecting lipid A biosynthesis and regulatory pathways, including *mgrB*, *pmrA*/*pmrB*, *crrB*, *arnC*, *lapB*, and *lpxM*, similar to other studies [9,55]. The absence of plasmid-mediated *mcr* genes in the present study isolates indicates that resistance to colistin in this setting arises mainly through adaptive chromosomal mechanisms rather than horizontal gene transfer [56,57]. However, the detection of few mutations in both colistin-resistant and colistin-susceptible isolates indicates that their presence alone is insufficient to infer causality, and functional studies are required to establish their phenotypic impact [9,58]. The coexistence of multiple mutations within these pathways suggests stepwise evolutionary adaptation under selective pressure, highlighting the capacity of *K. pneumoniae* to rapidly evolve resistance even to last-resort antimicrobials [59,60].

An additional clinically important observation is the detection of an isolate (Kp1256) carrying an expanded virulence-associated genomic repertoire within an MDR background. This isolate, recovered from a pus sample of a 30-year-old male presenting with a skin abscess, harbored multiple siderophore systems, immune evasion determinants, adhesion factors, and a complete type II secretion system, consistent with a hypervirulence-associated genotype. While these genomic features are often associated with hypervirulence-linked lineages, the presence of a single isolate is insufficient to infer the emergence or dissemination of convergent high-risk lineages. This finding should instead be viewed as hypothesis-generating, highlighting the potential for virulence-associated genetic traits to co-occur within MDR backgrounds and warranting further investigation in larger, longitudinal studies incorporating phenotypic virulence assays [12,61]. Such convergence challenges the traditional dichotomy between classical MDR and hypervirulent *K. pneumoniae* and has been increasingly reported worldwide, with significant implications for clinical management and patient outcomes [62,63].

Despite the strengths of this study, several limitations should be acknowledged. WGS was performed on a subset of MDR/XDR isolates and may not fully capture the genomic diversity of *K. pneumoniae* circulating in the region. Resistance-associated mutations and virulence determinants were identified through in silico analyses and were not functionally validated; therefore, they should be considered putative [19,64]. Additionally, phenotypic assays to confirm hypervirulence, such as string tests or in vivo infection models, were not performed. Finally, isolates were collected from a single geographic region, which may limit the generalizability of the findings.

## 5. Conclusions

This study provides a comprehensive genomic characterization of MDR and XDR *K. pneumoniae* in Peshawar and demonstrates that resistance in this setting is driven by a complex interplay between plasmid-mediated gene acquisition and chromosomal adaptive evolution. The identification of chromosomally mediated colistin resistance and virulence-associated genomic features within MDR backgrounds underscores the urgent need for sustained surveillance. WGS-based approaches are essential to inform infection control strategies, guide antimicrobial stewardship, and mitigate the spread of high-risk *K. pneumoniae* lineages in local contexts. However, the findings should be interpreted cautiously, as the study is based on a limited number of isolates from a restricted geographic area and may not be fully representative of *K. pneumoniae* circulating across Pakistan. Rather than supporting broad surveillance conclusions, these results provide a localized genomic snapshot and generate hypotheses that warrant validation through larger, multi-center genomic studies incorporating more comprehensive epidemiological data.

## Figures and Tables

**Table 1:** Chromosomal mutations associated with AMR in clinical *K. pneumoniae* isolates identified by WGS.

Gene	Protein/Function	Mutation(s)	Associated Resistance
*blaSHV*	β-lactamase	G259T, G50A, T290A, T47A	β-lactams
*CirA*	Siderophore receptor	E644V, E81V, V237I, Y183W	β-lactams (porin-related)
*FtsI (PBP3)*	Penicillin-binding protein	V192G, V375A	β-lactams
*LamB*	Maltoporin	G129D, K49Q	β-lactams
*OmpK35*	Outer membrane porin	A101S, Y108S	β-lactams
*OmpK36*	Outer membrane porin	D306N, D344E, E308ER, H349R, HN349RK, HN349RR, I315L, KL231IP, KL231VP, L184DEL, L307I, LGD225TDE, N221H, N276D, S346D, T192G, T258S, V178P, Y201F	β-lactams
*PBP3*	Penicillin-binding protein	D350N, S357N	β-lactams
*PmrA*	Two-component regulator	M66I	Colistin
*PmrB*	Sensor kinase	L213M	Colistin
*ArnC*	LPS modification enzyme	A249T, S30T	Colistin
*LapB*	LPS biosynthesis regulator	N212T	Colistin
*LpxM*	Lipid A acyltransferase	S253G	Colistin
*MgrB*	Negative regulator of PhoPQ	M1V	Colistin
*CrrB*	Sensor kinase	I127V, K325R, Q287K	Colistin
*ParC*	Topoisomerase IV	N304S, S80I, S681A	Fluoroquinolones
*GyrA*	DNA gyrase subunit A	E247K, F862I, S83I, N19S	Fluoroquinolones
*RamR*	Efflux regulator	I141T, M184V	Tetracycline
*RpsJ*	Ribosomal protein S10	K71L, S21A	Tetracycline
*EF-Tu*	Translation elongation factor	R234F	Pulvomycin
*UhpT*	Fosfomycin transporter	E350Q	Fosfomycin
*MarR*	Global transcriptional regulator	S3N	Ciprofloxacin, tetracycline

## Data Availability

The sequencing data generated in this study have been deposited in the NCBI Sequence Read Archive (SRA) under BioProject accession number PRJNA1377269.

## Supplementary Materials

The following supporting information can be downloaded at: https://www.mdpi.com/article/10.3390/pathogens15020218/s1, Table S1: Antibiotics used in the study, their abbreviations, disc concentrations, antibiotic classes and mechanism of action; Table S2: Characteristics of the 55 K. pneumoniae infections; Table S3: Genomic characteristics of the 16 K. pneumoniae sequenced isolates.
