# Supplementary material for: Genomic Insights into Chromosomal Colistin Resistance and Virulence–Resistance Convergence in MDR/XDR Klebsiella pneumoniae from Tertiary Hospitals in Peshawar, Pakistan
Source: Pathogens. 2026 Feb 14;15(2):218. doi: 10.3390/pathogens15020218 (PMC12943686; doi:10.3390/pathogens15020218)
Supplement: Supplementary file 1 [file pathogens-15-00218-s001.zip › pathogens-4129128-supplementary.pdf]

Supplementary Material

Table S1. Antibiotics used in the study, their abbreviations, disc concentrations, antibiotic classes and mechanism of action

Antibiotic	Abbreviation	Concentration (μg/disc)	Antibiotic Class	Role
Ampicillin	AMP	10	Penicillin	Cell wall synthesis Inhibitor
Amoxicillin–Clavulanate	AMC	20	β-Lactam/β-Lactamase inhibitor combination	
Amikacin	AK	30	Aminoglycoside	
Gentamicin	CN	10	Aminoglycoside	
Cefotaxime	CTX	30	Cephalosporin	
Ceftazidime	CAZ	20	Cephalosporin	
Cefepime	FEP	6.5	Cephalosprin	
Cefoperazone–Sulbactam	SCF	1.5	β-Lactam/β-Lactamase inhibitor combination	
Piperacillin–Tazobactam	TZP	4.5	β-Lactam/β-Lactamase inhibitor combination	
Ceftazidime–Avibactam	CZA	2.5	β-Lactam/β-Lactamase inhibitor combination	DNA Gyrase/Topoisomerase Inhibitor
Meropenem	MEM	10	Carbapenem	Protein Synthesis Inhibitor
Tigecycline	TGC	0.5	Tetracycline	
Ciprofloxacin	CIP	30	Floroquinolone	
Tetracycline	TET	30	Tetracycline	

Table S2. Characteristics of the 55 *K. pneumoniae* infections

Characteristic	N	%
Age (years) (median and Range)	(33.4)	(21-47)
Female	30	54.55
Male	25	45.5
Location		
Mardan	20	36.36
Takht Bhai	13	23.64
Peshawar	10	18.18
Swabi	8	14.5
Charsadda	4	7.27
Infection types		
Urinary Tract Infections	13	23.64
Abscesses	12	21.81
Skin infections	9	16.36
Bacteremia	8	14.5
Respiratory tract infections	7	12.72
Wounds	6	11
Place of treatment		

General Surgery	15	27.27
Internal Medicine	14	25.45
Orthopedics	11	20.00
Intensive care unit	8	14.55
Pediatrics	7	12.73
AST resistance		
AMP	55	100
AMC	28	50.9
AK	14	25.45
CN	22	40.00
CTX	29	52.73
CAZ	21	38.18
FEP	20	36.36
SCF	12	21.82
TZP	10	18.18
CZA	27	49.09
MEM	11	20.00
TGC	7	12.73
CIP	20	36.36
TET	39	70.91
Resistance to polymyxins (colistin)	8	14.54
Susceptible	26	47.27
MDR	19	34.55
XDR	10	18.18
Virulence genes per isolate	(16)	(9-75)
Yersiniabactin	11	68.75
Enterobactin	2	12.50
Effector proteins (espL1, espR1)	1	6.25
Fimbriae	1	6.25
Type II secretion system	1	6.25

Table S3: Genomic characteristics of the 16 *K. pneumoniae* sequenced isolates

Sample ID	Contig Count	Largest Contig	Total Size (bp)	L50	N50	tRNA	rRNA	CDS	GC Content (%)
1218	148	366,030	3,990,291	10	136,424	63	6	3,895	57.12
942	193	1,073,600	5,491,155	5	350,969	84	17	5,440	57.24
1231	240	419,845	5,626,701	10	220,756	83	11	5,646	57.12
1229	212	419,698	5,621,746	10	216,442	87	13	5,620	57.14
1226	221	420,007	5,621,275	8	275,827	85	11	5,636	57.14
1227	399	493,342	11,944,267	17	239,143	1138	18	11,967	62
1202	258	435,873	5,958,915	10	214,758	82	13	6,078	56.68
1216	251	492,459	5,957,613	10	214,633	84	15	6,061	56.67
1217	745	300,466	6,298,127	20	107,582	98	17	6,741	55.51

1209	97	941,060	5,411,488	4	453,136	84	9	5,301	57.29
1213	95	941,060	5,410,365	5	395,886	84	9	5,298	57.29
1212	88	859,020	5,479,812	6	352,802	81	12	5,341	57.19
1205	218	1,619,838	5,690,287	4	406,469	83	14	5,665	57
1206	218	981,321	5,556,869	4	501,921	88	19	5,502	57.26
1256	403	513,349	10,237,651	19	146,129	173	29	10,232	54.33
1207	173	858,998	5,489,782	5	398,981	87	15	5,388	57.3
